# Commentary: Outlining a novel psychometric model of mental flexibility and affect dynamics

**DOI:** 10.3389/fpsyg.2025.1463888

**Published:** 2025-02-21

**Authors:** Thea Ionescu, Hippolyte Gros

**Affiliations:** ^1^Department of Psychology, Babeş-Bolyai University, Cluj-Napoca, Romania; ^2^Aix Marseille University, CNRS, CRPN, UMR 7077, Marseille, France

**Keywords:** cognitive flexibility, property, executive functions, conceptualization, affect

## 1 Introduction

The article “Outlining a novel psychometric model of mental flexibility and affect dynamics” by Borghesi et al. has the bold aim of reaching a unitary view on a construct that remains poorly understood (Ionescu, 2012; Müller and Kerns, 2015). They aptly highlight the multiple views in the literature, especially referring to cognitive flexibility as set-shifting (Diamond, 2006, 2013) and to flexibility as being present in the moment or expressing one's true self in acceptance and commitment therapy (ACT, Levin et al., 2017). These diverse perspectives certainly create confusion when exploring the literature and impair adequate psychological interventions (Ionescu, 2022). The authors propose unifying these views under “mental flexibility” and linking it to affect dynamics. However, their attempt has some drawbacks that might inadvertently contribute to the multiplication of views on flexibility. Below, we outline these issues.

## 2 A courageous endeavor and some problems

Borghesi et al. (2023) aim “(…) to disentangle the puzzle of flexibility by outlining the distinctive cross-domain features of this concept, thus providing a novel comprehensive operationalization” (p. 1). But two problems impede their endeavor.

*First*, coming from the affective domain, the authors sometimes mix ideas about cognitive flexibility and its measurements. They observe that cognitive flexibility is more prominent in the literature than flexibility in general, especially in research on executive functions where it is considered an ability. However, their claim “Moreover, Ionescu (2012) already hypothesized that cognitive flexibility, *identified only as a neurological function related to executive functions*, might be a shared feature of different processes” (p. 03, italic added) is incorrect. The cited work aimed to go beyond what the executive functions literature was offering. As such, there are already attempts to view flexibility as a property of cognitive functioning (Clément, 2009; Deak, 2003; Ionescu, 2012). But while the authors acknowledge that flexibility may be a property, they also use the terms “mechanism” (p. 1…), “skill” (p. 2, 5), and “ability” (p. 1–5, 7, 10, 12…), confusing the reader. Specifically, if flexibility is viewed as an ability or skill, it implies that it is a stable entity in the cognitive system, while if it is considered a mechanism, it suggests a well-delineated piece of such a fixed entity. On the other hand, conceptualizing flexibility as a property suggests that any process, be it cognitive or not (i.e., it could also be affective) may reach functioning flexibly in certain conditions (Ionescu, 2017). As such, we no longer need to infer a distinct entity anymore, namely some “flexibility module” that adds to other abilities or mechanisms. What is important however is to be clear about the superordinate category in which we include flexibility: a fixed entity or an emergent property.

Similarly, the authors' ambition to account for cognitive and conative features in flexibility seems limited by insufficient engagement with the literature. For instance, **Table 1** lists the “Measurements of mental flexibility,” but only provides a partial overview of the vastly diverse measures reported in the literature. Considering the numerous approaches tackling flexibility, each using different tasks to evaluate specific forms, any unified framework should start by identifying the common components targeted by those tasks. However, the authors omit several crucial tasks (e.g., the *DCCS*, the *Brixton task*, the *Navon task*, the *plus minus task*, the *Innovative paradigm*, see Maintenant and Bodi, 2022 for a review). Thus, their psychometric model may only account for a very limited scope of flexible behaviors. Additionally, their exclusive reliance, during data collection, on self-report measures of flexibility which assess a different construct than direct measures (Howlett et al., 2022), weakens their argument for a comprehensive model of “mental flexibility.”

*Second*, adding “mental” to flexibility is not new: many articles on cognitive flexibility as set-shifting use it synonymously (Anziano et al., 2023; Dibbets and Jolles, 2006). The authors propose that adding “mental” before flexibility allows to “study flexibility unambiguously within the psychological domain” (p. 3). However, many ambiguities remain with regards to what this construct would entail. Moreover, their argument of this term not “implying any connection with physics or materials chemistry” (p. 3) is difficult to grasp in an era when neuroscience helps us decipher the link between the body and cognitive and affective aspects of our mental world (Damasio, 2019), especially because the authors themselves want to include affect dynamics under this construct.

## 3 Variability or flexibility?

Another puzzling idea is that of flexibility being “adaptive variability,” especially since the authors also claim it “dwells between variability and adaptivity” (p. 4). When presenting in **Figure 2** flexibility as “adaptive variability” property, the authors argue that “To adapt to different environmental and social demands, individuals may feel pressure to change their behaviors or values in ways that are not consistent with their true selves, so they are constantly adapting to new situations and shifting their behaviors or values without taking responsibility for their actions (**Figure 2**; O'Toole et al., 2020; Chen and Tang, 2022)” (p. 3). However, neither O'Toole et al. (2020) nor Chen and Tang (2022) defend that adapting to a changing environment implies refusing responsibility or inconsistencies with one's “true self.” Considering the subtleties between the different accounts of variability and flexibility, it seems important to remain as close as possible to the authors' intended meaning.

While from a dynamic systems point of view one could envisage behavior moving from instability to stability and back, viewed as flexibility—stability—flexibility by some (Defeyter and German, 2003; Gopnik et al., 2017), it seems more logical to see this as going from variability (changing behavior without necessarily knowing how) to stability (well-known behaviors for well-known problems) to flexibility (changing behavior to find new solutions) (Ionescu, 2017). For example, in language development one can see a toddler changing verbal labels for a seen object until the adult confirms the new word (naming an excavator as dump truck, garbage truck, and then excavator—reflecting variability); then we see the toddler using correctly the new word for the proper object (always naming appropriately the excavator—reflecting stability); and finally, we see the toddler naming with several appropriate labels the same object (like excavator and construction truck—reflecting flexibility). Further studies are needed to confirm this pattern, but some lines of work on executive functions do lend indirect support by acknowledging the important role of goal monitoring for flexibility to appear (Chevalier and Blaye, 2016, 2022). Thus, it is hard to equate variability and flexibility without supporting data.

## 4 Discussion

Nevertheless, the authors' idea to apply Markov chains to model flexibility is intriguing since it allows for the representation of cognitive processes as state transitions, potentially capturing the dynamic nature of cognitive flexibility, where the mind shifts from one mode of thinking to another. This could help create a quantitative framework to measure flexibility and enable predictive modeling. However, the current model has serious limitations due to its reliance on a flawed definition of flexibility and fragmentary measurements. Refining the model and validating it with adequate empirical data could enhance our understanding of cognitive flexibility and its underlying mechanisms. A major step in achieving this is to be as precise as possible about the definition of flexibility and then choose the measures accordingly. While we acknowledge the challenge posed by the fragmented literature on flexibility, we encourage the authors to very carefully consider the nature of flexibility before attempting to model and test it.

If we are to foster flexibility during ontogeny, to help individuals who may lack it or to stimulate innovation and creativity, we need more clarity in the field so that we can design efficient interventions for flexibility development.
